# Upstream process optimization and its critical role in chimpanzee adenoviral vector clinical drug development: lessons learned from VRON-0200 manufacturing

**DOI:** 10.3389/fbioe.2026.1934410

**Published:** 2026-09-10

**Authors:** Larissa H. Haut, Janice Bennett, Paula MacDonald

**Affiliations:** Virion Therapeutics, Philadelphia, PA, United States

**Keywords:** adenoviral vector manufacturing, checkpoint modifier, chimpanzee adenovirus vector, chronic hepatitis B, functional cure, therapeutic vaccine, upstream process optimization, VRON-0200

## Abstract

Chimpanzee adenoviral (AdC) vectors represent a powerful platform for immunotherapies due to their strong immunogenicity, excellent safety profile, low human seroprevalence, and minimal cross-neutralization to prevalent human serotypes. Despite these advantages, AdC manufacturing presents unique challenges, including elevated infectivity ratios, transgene-dependent replication kinetics, and higher impurity burdens compared to well-characterized human adenovirus systems. During VRON-0200 development, a first-in-class heterologous AdC-based immunotherapy targeting functional cure in chronic hepatitis B, we found that upstream process optimization, rather than downstream purification, is the main driver of crude harvest quality and final drug product attributes. Iterative refinement of permissive cell growth cycles, time of harvest, and multiplicity of infection, guided by a robust, tiered analytical strategy, delivered high-purity harvests with superior infectivity ratios, reduced host cell residuals, minimized product-related impurities, and diminished downstream processing demands. These manufacturing refinements supported GMP production and Phase 1b clinical outcomes demonstrating safety, tolerability, and significant anti-HBV activity in chronically infected patients. We contend that the bioprocessing community’s longstanding emphasis on downstream polishing overlooks the foundational role of upstream control in addressing AdC-specific hurdles. Reprioritizing upstream-first strategies holds transformative potential to improve vector quality, enhance safety profiles, accelerate development timelines, and increase cost-effectiveness for AdC and related vector platforms.

## 1 Introduction

Chronic hepatitis B virus (HBV) infection affects approximately 257 million individuals globally (World Health Organization, 2026), which can lead to lifelong health consequences, including cirrhosis, liver failure, hepatocellular carcinoma, and death. Current standard-of-care nucleos(t)ide analog therapies (e.g., tenofovir, entecavir) effectively suppress viral replication and slow disease progression but require lifelong administration and rarely result in functional cure, defined as sustained undetectable HBV surface antigen (HBsAg) and serum HBV DNA after completing a finite course of treatment. Achieving functional cure is widely recognized to require immune modulators to restore the host’s exhausted immune responses, as antiviral suppression alone is typically insufficient for sustained viral control. As such, novel immunotherapeutic approaches that are safe, convenient, and able to restore robust, virus-specific immunity are needed.

VRON-0200 was designed to address this unmet medical need: a therapeutic immunotherapy comprising two heterologous, replication-defective AdC vectors (AdC6-gDHBV2 and AdC7-gDHBV2) expressing highly conserved HBV core and polymerase antigens genetically fused to herpes simplex virus-1 glycoprotein D (gD) (Hasanpourghadi et al., 2021). This fusion serves as a novel molecular checkpoint modifier that lowers the T cell activation threshold, thereby enhancing and broadening CD8^+^ T cell responses against HBV. Phase 1b clinical results have demonstrated that VRON-0200 was safe and well-tolerated, inducing durable HBV-specific immune restoration in nucleos(t)ide-treated chronically infected patients after a single intramuscular administration. Sustained or deepening HBsAg declines were observed in the majority of patients during 2-year follow-up after prime dose (Gane et al., 2026). Manufacturing and clinical development planning are underway to progress VRON-0200 for HBV functional cure studies.

AdC vectors offer compelling advantages over human adenovirus serotypes for therapeutic vaccination. They exhibit high intrinsic immunogenicity without requiring adjuvants (Tatsis and Ertl, 2004), display low seroprevalence in human populations, and demonstrate limited cross-neutralizing immunity to common human adenovirus serotypes (Chen et al., 2010). AdC6 and AdC7 vectors, engineered with E1 deletion and partial E3 retention, are produced in E1-trans-complementing cell lines (e.g., HEK293 derivatives) and show enhanced genetic stability and transgene expression for challenging antigens (Haut et al., 2016). However, AdC platforms present distinct manufacturing considerations from those of extensively studied human adenovirus serotype 5 vectors. These include higher baseline infectivity ratios (total viral particles [VP] per infectious units [IU]) (Hickey et al., 2023), greater sensitivity to cell line passage effects, and tendency toward elevated product-related impurities (empty or immature capsids, free viral proteins). These platform-specific attributes are routinely addressed through targeted process optimization to maintain product quality and acceptable immunogenic risk profile, considerations that are inherent to every adenoviral platform, each with its own unique manufacturing nuances.

While industry practice and published literature frequently prioritize downstream purification strategies (e.g., ion-exchange chromatography, tangential flow filtration, size-exclusion steps) (Farnós et al., 2023; Peixoto et al., 2008; Wu et al., 2025) as the principal means to achieve drug product (DP) purity and potency, our hands-on experience in the process development and GMP manufacturing of VRON-0200 suggests otherwise. Upstream process parameters emerged as the dominant, often decisive, factor shaping crude harvest quality, which in turn set fundamental limits on final DP attributes that downstream operations could only partially mitigate. Suboptimal upstream conditions generated co-purifying impurities, accelerated vector degradation during processing, and reduced overall recovery yields. In contrast, rigorous upstream optimization produced exceptionally clean crude harvests that required minimal downstream intervention while preserving infectivity, capsid integrity, and low impurity profiles. We propose that this upstream-centric paradigm represents an opportunity to overcome persistent bottlenecks in AdC vector production.

## 2 Upstream parameters as the dominant influence

In viral vector biomanufacturing, the upstream process establishes the initial quality landscape. Crude harvests burdened with host cell proteins, residual DNA, unpackaged viral genomes, empty or incomplete capsids, and aggregated particles impose severe constraints on downstream purification efficiency (Ahi et al., 2011; Thacker et al., 2009). Managing the impurity profile of AdC vectors is critical, as elevated levels of free viral components or non-infectious particles can prime innate and adaptive immune signaling. Such interactions may broaden the reactogenicity of the vector, potentially impacting the overall inflammatory baseline in a clinical setting.

Our experience identified three critical, and interconnected, upstream factors in the VRON-0200 development process: cell maintenance and growth cycle management, time of harvest (TOH), and multiplicity of infection (MOI), whose optimization was enabled by a fit-for-purpose analytical monitoring strategy.

Permissive cell maintenance and growth cycle management provided the foundation for reproducible, high-productivity infections. HEK293 derivatives used for AdC production exhibit passage-dependent changes in growth rate, morphology, and viral replication competence. The effects of cell culture growth kinetics extend beyond limitations related to the viral yields targeted during large-scale manufacturing. Sub-optimal culture conditions reduce cell growth efficiency, necessitating larger culture volumes to achieve the desired total cell mass and viral output. These larger volumes result in higher levels of cellular components in the crude harvest matrix, leading to more challenging downstream processing and potential reductions in viral recovery post-clarification due to physical entrapment, adsorption, or degradation of VPs. By establishing tight control over subculture timing, seeding densities, and passage limits, we minimized lag phases, maximized viable cell mass at infection, and achieved predictable seed train expansions across scales. Infection during early-to-mid exponential growth was critical to prevent cell entry into stationary phase prior to or during viral replication, thus yielding higher productivity with lower residual profiles (e.g., host cell DNA and proteins, defective or incomplete VP). When infections were initiated at later timepoints, during the mid to late exponential or stationary phase, they consistently produced lower infectious titers and higher cellular residuals, likely attributable to the suboptimal metabolic state of the host cells.

TOH emerged as the single most influential upstream parameter. Harvesting prematurely yields unpackaged or immature particles, whereas harvesting beyond peak maturation increases turbidity, host cell protein carryover, and viral aggregation. Establishing an optimal TOH required construct- and backbone-specific time-course studies that balanced capsid assembly, genome packaging, and maturation against the risk of excessive cytolytic activity and release of intracellular contaminants. Although optimal processing parameters for distinct adenovirus serotypes can vary due to intrinsic serotype-related differences, results from development studies showed that AdC6 and AdC7 demonstrate comparable infection, harvest, and purification kinetics. Given that downstream processing was otherwise identical for both vectors, discrepancies sometimes observed in DP quality were noted to correlate with upstream parameter variability. Even small differences in pre-lysis cell viability between AdC6 and AdC7 harvests led to measurable DP quality differences: higher viability at harvest correlated with cleaner matrices, lower residual host cell proteins, higher full-capsid percentages (by analytical ultracentrifugation [AUC]), and reduced aggregation, despite identical downstream processing (Table 1). Immediate post-harvest lysis, pH adjustment, and clarification were found to be critical to preserve infectious titer and prevent quality degradation during hold steps. This strategy precluded downstream carryover of contaminants such as non-infectious VPs that frequently exhibit physicochemical similarities to the target material, and could result in their co-purification or incomplete removal in the final product despite advanced chromatographic or filtration techniques.

**TABLE 1:** Representative effects of upstream parameter variability on drug product quality. Data show correlation between viral harvest viability (infected culture viability pre-lysis) and DP quality attributes during process development.

Parameter	AdC6	AdC7
Seed train viability (%)	98.1	96.9
Satellite pre-infection viability (%)	98.3	97.2
Harvest pre-lysis viability (%)	87.5	92.0
Post-lysis turbidity (NTU)	19.7	14.8
Post-clarification turbidity (NTU)	2.23	2.23
Lysed bulk harvest infectivity ratio (VP:IU)	535	423
DP infectivity ratio (VP:IU)	176	356
DP AUC	9.9% HOC 72.0% full capsid	1.3% HOC 80.9% full capsid

**FIGURE 1:**
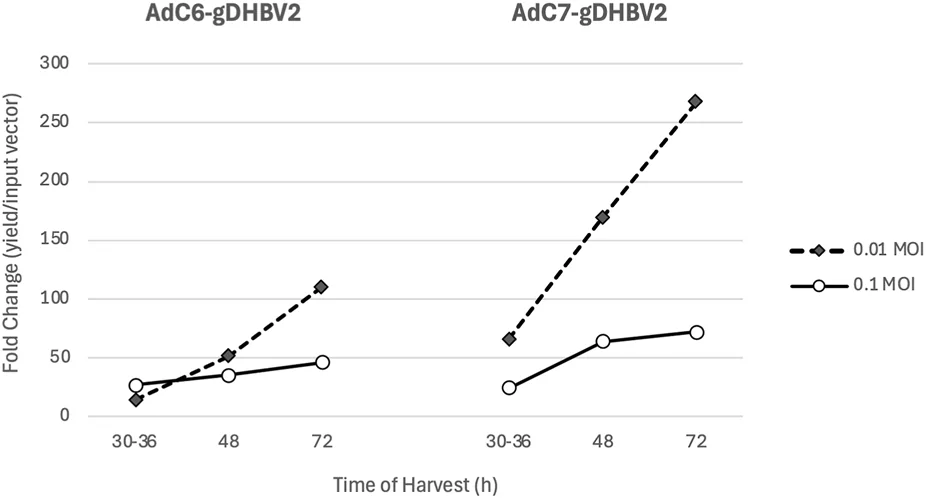
Representative TOH and MOI kinetics for VRON-0200 vectors. Data are shown as fold change (IU values expressed as harvest yield per input vector) for two MOIs at three harvest timepoints.

MOI determination required careful balancing of virus inoculum concentration, TOH, and cell density at infection to optimize replication kinetics, per-cell productivity, and harvest purity. High MOIs accelerated replication but often led to premature cell lysis, culture saturation, and reduced yields per input vector. Low MOIs (<1, based on immunostaining) with extended incubation allowed two full replication cycles per viral passage (Figure 1), enhancing fidelity, increasing full-capsid content, and lowering impurity burdens. While extended cycles raised theoretical recombination risks, these were mitigated through expanded quality testing including next-generation sequencing and absence of wild-type variants.

Suboptimal upstream conditions often yield crude harvests with elevated impurity profiles, such as host cell proteins, nucleic acids, and non-infectious VPs, that impose inherent limitations on downstream purification efficacy. Consequently, upstream deficiencies cannot be fully mitigated by downstream operations alone, as prolonged exposure to existent processing conditions or the incorporation of additional purification steps exacerbates vector degradation, reduces recovery yields, and compromises final product potency and safety metrics. Taken together, these factors underscore the benefits of an upstream-centric manufacturing strategy for overall process robustness, as evidenced by our observation that the combination of low MOI, growth-cycle-aligned infection, and optimized TOH consistently generated crude harvests with markedly improved quality attributes.

## 3 The pivotal role of analytical monitoring

Informing upstream decisions demanded a fit-for-purpose, evolving analytical strategy. Early reliance on immunostaining provided rapid infectious titer feedback for screening, while ddPCR offered absolute, matrix-robust genomic titer quantification across crude-to-purified samples. Infectious titer evaluation transitioned to TCID_50_ with ddPCR endpoint readout for greater precision, especially given AdC vectors’ sometimes subtle cytopathic effects. Rapid full/empty screening via the Stunner platform (UV/Vis + light scattering) enabled timely optimization during development, with AUC serving as the gold-standard orthogonal method for final DP capsid population characterization.

Infectivity ratio (VP:IU) alone proved inadequate as a quality surrogate (Table 1); uncorrelated orthogonal readouts of capsid integrity, aggregation, and host cell residuals revealed persistent differences attributable to upstream conditions.

In addition to key analytical product parameters such as VP, IU, capsid population, and vector identity, upstream operations were monitored in real-time with qualified assays for standard process parameters (pH, dissolved gases, key metabolites, etc*.*) alongside dedicated tests for process-related residuals (host cell DNA and proteins, Benzonase, and other contaminants). This testing strategy resulted in an integrated, data-rich monitoring framework that transformed empirical trial-and-error into precise, evidence-based parameter refinement.

## 4 Discussion

The bioprocessing field has long treated downstream operations as the primary quality gatekeeper for viral vectors. Yet upstream deficiencies, once embedded in the harvest matrix, frequently result in co-purification of like-sized or physicochemically similar impurities, vector instability during extended processing, and irrecoverable potency losses. For AdC vectors, where minimizing total particle load and product-related impurities that drive inflammatory responses is critical, upstream optimization may offer a more direct and efficient path to high-quality material.

VRON-0200 experience yields the following key principles for AdC vector manufacturing:

- Prioritize upstream steps and high-purity crude harvests in process design rather than relying solely on downstream polishing to ensure DP quality and safety.
- Align development studies rigorously with intended GMP passage numbers, and scale-specific kinetics to ensure translatability.
- Strategically balance MOI, TOH, and cell growth phase to maximize viral replication fidelity, packaging and maturation efficiency, and impurity control.
- Implement hybrid analytical platforms combining rapid screening tools with high-precision orthogonal methods for timely, robust decision-making.

AdC vector development suggests that integrating process analytical technologies (PAT) to enable real-time upstream control could further strengthen process robustness. Rigorously validated low-MOI, extended-TOH protocols may provide a cost-effective approach to high-quality production for AdC and related platforms designed for chronic infections and other diseases such as cancer.

Experience gained during upstream process optimization for VRON-0200 manufacturing demonstrates that prioritizing crude harvest quality reduces downstream complexity, increases yields, and better preserves critical quality attributes. Focusing on upstream operations mitigates viral vector manufacturing bottlenecks, and not only has the potential to accelerate clinical translation but also to enhance the safety and efficacy of therapies in development.

## Data Availability

The datasets presented in this article are not readily available because they include commercially sensitive information, and are available upon reasonable request from the corresponding author. Requests to access the datasets should be directed to Larissa Haut, lhaut@viriontx.com.
